# The effect of ultrasound-guided thoracic paravertebral nerve block combined with hydromorphone on postoperative analgesia and inflammatory response in thoracoscopic surgery: a randomized controlled trial

**DOI:** 10.3389/fmed.2025.1587477

**Published:** 2025-07-25

**Authors:** Lin Zeng, Xiaoxue Yu, Ting Yang, Jing Liao, Yinghui Ouyang

**Affiliations:** Department of Anesthesiology, Shifang People’s Hospital, Shifang, Sichuan, China

**Keywords:** analgesia, ultrasound-guided thoracic paravertebral block, thoracoscopic surgery, hydromorphone, inflammation

## Abstract

**Objective:**

This study aims to explore the effects of a multimodal postoperative analgesia regimen of ultrasound-guided thoracic paravertebral block (UTPB) combined with hydromorphone patient-controlled intravenous analgesia (PCIA) on postoperative analgesia, early recovery, and traumatic inflammatory response in patients undergoing thoracoscopy surgery, providing a basis for clinical application.

**Methods:**

In this single-blind, randomized controlled trial (RCT), we enrolled 64 patients scheduled for thoracoscopic lobectomy of the lung. The patients were randomly assigned into two groups of UTPB combined with hydromorphone PCIA group (T group) and hydromorphone PCIA group (H group) according to the random number table method. T group underwent UTPB with ropivacaine before induction of general anesthesia. H group is the control group. Both groups underwent PCIA after surgery, with the analgesic formula being 50 μg/kg of hydromorphone. The analgesic solution is prepared with 100 mL of sodium chloride injection, a background dose of 2.0 mL/h, a patient-controlled analgesia dose of 2 mL, and a locking time of 30 min.

**Results:**

Compared with H group, T group showed a significant decrease in visual analog scale (VAS) scores and an increase in bruggrmann comfort scale (BCS) scores within 8 h after surgery, and a decrease in opioid analgesic doses during and within 8 h after surgery. The levels of serum C-reactive protein (CRP), Interleukin-6 (IL-6), and tumor necrosis factor (TNF-α) in T group were lower than those in H group at 24 h after surgery. The first time patients in T group got out of bed after surgery was earlier than that in H group. Two groups of patients had no serious adverse reactions.

**Conclusion:**

Ultrasound-guided thoracic paravertebral block combined with hydromorphone PCIA can achieve good analgesic effects in thoracoscopic lobectomy surgery. It can reduce the amount of opioid drugs used during the perioperative period, alleviate the body’s inflammatory response, and promote rapid recovery of patients. It has clinical application value.

**Clinical trial registration:**

https://www.chictr.org.cn/showproj.html?proj=201650, identifier ChiCTR2300074082.

## 1 Introduction

Thoracoscopic surgery is the representative operation of minimally invasive thoracic surgery in the treatment of lung cancer and is also the development direction of thoracic surgery in the future (1). Compared with thoracotomy, thoracoscopic surgery has the advantages of less surgical trauma, less postoperative pain, less impact on lung function, less impact on immune function and less postoperative complications (2). Although the incision of thoracoscopic surgery is small, many patients after thoracoscopic surgery still have obvious pain (3). Early studies reported that the incidence of pain in patients undergoing thoracoscopic lobectomy was 36% (4). The rate of chronic pain in patients undergoing thoracoscopic surgery in the Netherlands is as high as 47% (5). The methods of relieving postoperative pain in clinical anesthesia include intraspinal analgesia,(6) intercostal nerve block analgesia,(7) paraspinal block analgesia,(8) and erector spinae muscle block analgesia (9). Opioids are widely used in clinic as analgesics that act on μ, κ, and δ receptors in the brain, peripheral nervous system and spinal cord to produce powerful analgesic effect (10). Previous studies have shown that pain can trigger the activation of glial cells, mediate neuroinflammation, and increase endogenous pro-inflammatory cytokines (11, 12). The increase of proinflammatory cytokines will reduce the function of neurons (13).

Ultrasound visualization technology has become an indispensable part of modern medicine. Ultrasound-guided thoracic paravertebral block (UTPB) is more and more widely used in thoracoscopic surgery (14, 15). Thoracic paravertebral block (TPB) is a technique of injecting local anesthetics into the thoracic paravertebral space through puncture, which is similar to the effect of unilateral epidural block (16, 17). This effectively reduces postoperative incision pain, catheter irritation pain, and visceral pain (18). Current research indicates that paravertebral block can reduce the incidence of hypotension compared to thoracic epidural anesthesia (19).

Hydromorphone is a semi synthetic μ and δ opioid agonist that widely used in European and American countries. Hydromorphone has valuable advantages such as rapid onset, strong analgesia, unlimited analgesic effect, no pharmacological activity of metabolites, and fewer side effects (20–22). Sufentanil, an analog of fentanyl, has the advantages of good analgesic effect and less side effects (23, 24). Studies have reported that hydromorphone has the advantage of improving patients’ mood compared with sufentanil (25). Hydromorphone has a high affinity for both μ opioid drugs and δ opioid receptors. Studies have found that the use of hydromorphone can improve the postoperative mood of patients because μ opioid receptor excitation can relieve pain and δ opioid receptor excitation can relieve anxiety (26).

After investigation, it was found that there is limited research on the impact of thoracic paravertebral nerve block on surgical stress response. Postoperative pain can also trigger the body’s stress response, leading to the release of inflammatory factors that can cause non-specific degeneration of damaged nerve endings, resulting in hyperalgesia (27–29). Preoperative prophylactic analgesia can reduce incision pain and inflammatory pain, as well as decrease peripheral and central sensitization. Neural blockade can significantly reduce the secretion of inflammatory factors by adequately relieving pain and suppressing stress responses (30). So the role of multimodal analgesia combined with multiple administration routes in enhanced recovery after surgery (ERAS) is still the main research direction.

## 2 Methods and materials

### 2.1 Study design

Inclusion criteria: (1) all patients underwent thoracoscopic lobectomy; (2) American Society of Anesthesiologists (ASA) class I-III. Exclusion criteria: (1) patients with mental illness; (2) drug or alcohol addicts; (3) complicated with pleural thickening or calcification; (4) tuberculosis patients; (5) severe liver and kidney dysfunction; (6) coagulation dysfunction.

The patients participating in the study were randomly divided into T group and H group. Randomized sequences are generated by computers and hidden in opaque sealed envelopes. On the day of the surgery, an anesthesiologist opened the envelope to determine the grouping. After the anesthesia is administered, another anesthesiologist is responsible for observing and following up on the patient’s indicators. The surgery and anesthesia in this study were performed by the same group of doctors.

### 2.2 Anesthetic protocol

After entering the operating room, patients undergo routine monitoring, including electrocardiogram (ECG), pulse oximetry (SPO2), heart rate (HR), non-invasive arterial blood pressure (NIBP), and respiratory rate (RR). Patients in T group underwent thoracic paravertebral block with 0.33% ropivacaine 30 ml before induction of general anesthesia. Under sterile conditions, the anesthesiologist places a linear ultrasound probe (high frequency 6 - 13 MHz; Mindray, China) between the 5th and 6th thoracic spinous processes. After local anesthesia, a 10 cm 22 g nerve stimulation block needle (Pajunk, Germany) is inserted using a planar technique and guided by ultrasound to puncture the paraspinal space. After confirming the needle tip position, ropivacaine is administered. H group was the control group. The anesthesia induction drugs for both groups of patients were sufentanil 0.5 μg/kg, propofol 2 mg/kg, and rocuronium bromide 0.6 mg/kg. After the anesthetic was fully effective, a double lumen bronchial tube was inserted under the guidance of a laryngoscope, and then a fiberoptic bronchoscope was used to check and ensure that the tube was properly aligned before fixing it. Two groups of patients received mechanical ventilation through a ventilator with an oxygen flow rate of 2.0 L/min, a tidal volume of 6–8 mL/kg, and a respiratory rate of 12–16 times/min. Two groups of patients received inhalation of 1%–2% sevoflurane and micro pump infusion of 0.1–0.3 μg/kg/min remifentanil to maintain anesthesia during surgery (31). During the operation, respiratory parameters were adjusted to maintain the end expiratory carbon dioxide (PETCO2) at 35–45 mmHg. The monitoring index for anesthesia depth, bispectral index (BIS) of electroencephalography, was maintained at 40–60. After the operation, if the patient has indications for extubation, the double lumen bronchial tube will be removed and transferred to the anesthesia recovery room. Both groups underwent patient-controlled intravenous analgesia (PCIA) after surgery, with the analgesic formula being 50 μg/kg of hydromorphone (trade name: Ruining. Yichang Renfu Pharmaceutical Co., Ltd., specification: 2 mg: 2 mL). The analgesic solution is prepared with 100 ml of sodium chloride injection, with a background dose of 2.0 mL/h, a patient-controlled analgesia (PCA) dose of 3 mL, and a locking time of 15 min.

### 2.3 Blood samples collection and time points

2 ml of peripheral venous blood was collected from the patient before and 1 day after surgery and was centrifuged at 3000 r/min for 10 min at room temperature. Collect the supernatant of the sample after centrifugation and store it in a −80°C freezer.

### 2.4 Outcome measures

(1) General information such as age, gender, body mass index (BMI), operation time, infusion volume, blood loss and length of hospital stay.

(2) Visual analog scale (VAS) scores, bruggrmann comfort scale (BCS) scores, and PCA frequency of two groups of patients at 4 h (T1), 8 h (T2), 12 h (T3), 24 h (T4), and 48 h (T5) after surgery.

(3) Using enzyme-linked immunosorbent assay (ELISA) following manufacturers instructions, the inflammatory reaction index of C-reactive protein (CRP), tumor necrosis factor (TNF-α) and Interleukin-6 (IL-6) were compared between the two groups.

(4) Complications: bradycardia, hypotension, nausea, vomiting, and time of first postoperative mobilization.

### 2.5 Statistical analysis

According to the pre-experimental results, the sample size calculation was based on the VAS scores at T1 as the primary outcome. Set α = 0.05 and power = 0.8 was used to determine the study. According to the formula estimate the effective sample size N ≈ 23 for each group. Invasive clinical trials often need to recruit more subjects to prevent patients from falling out during follow-up. Based on experience, it is assumed that 20% of the trials are lost to follow-up, so each group of samples is adjusted up to N* = N÷(1−0.2) ≈ 29.

Data analysis was achieved by using SPSS for Windows version 26.0. Normally distributed measurement data were expressed as mean ± standard deviation ($\bar{\text{x}}$ ± s). Continuous variables that were normally distributed were analyzed using an independent *t*-test, while non-normally distributed variables were analyzed using the Mann–Whitney U test for comparison between the groups. Repeated measures analysis of variance will be used for intra-group comparisons. The χ^2^ test will be used for the comparison of count data. *P* < 0.05, indicating that the difference is statistically significant.

## 3 Results

### 3.1 Participants

**FIGURE 1:**
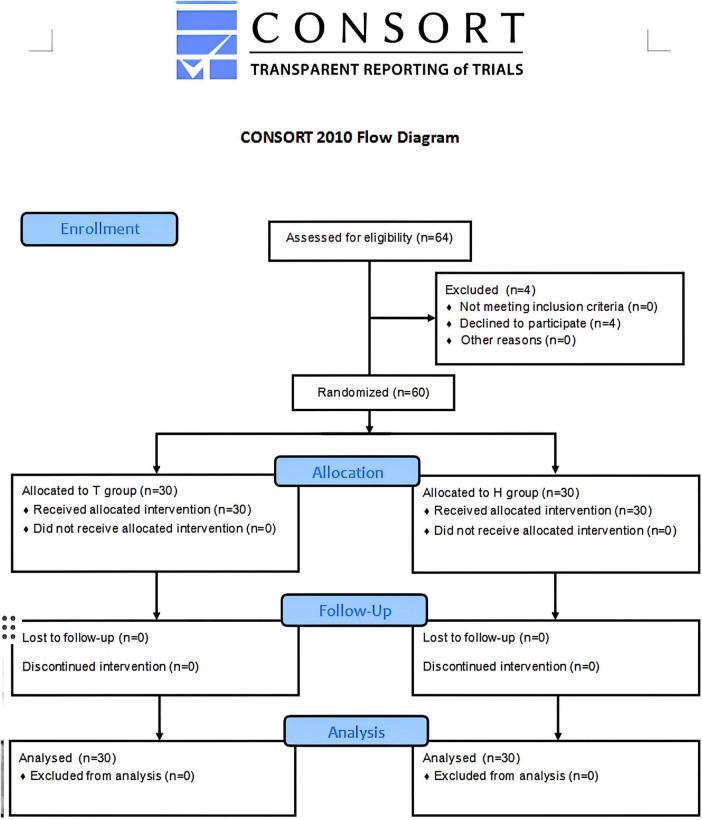
Consort diagram. Initially, 64 patients were randomly assigned to 1 of 2 groups as follows: thoracic paravertebral nerve block combined with hydromorphone postoperative analgesia group (T group) or hydromorphone postoperative patient-controlled analgesia group (H group). 60 patients (30 in T group and 30 in H group) completed this study.

A total of 64 patients who were scheduled for thoracoscopic lobectomy of the lung at Shifang people’s Hospital were enrolled in this study from July 2023 to December 2024. After 4 refused to participate, 60 eligible participants were included in the final analysis. They were randomly divided into thoracic paravertebral nerve block combined with hydromorphone postoperative analgesia group (T group) and hydromorphone postoperative patient-controlled analgesia group (H group) (Figure 1).

### 3.2 Demographic and perioperative data

As shown in Table 1, there was no significant difference in age, gender, BMI, operation time, infusion volume, intraoperative blood loss and hospitalization time between the two groups (*P* > 0.05).

**TABLE 1:** Demographic data in the study groups.

Index	T group(*n* = 30)	H group(*n* = 30)	*P-*value
Age (years)	56.53 ± 13.27	59.03 ± 13.57	0.474
Body mass index (kg/m2)	23.12 ± 3.01	22.81 ± 3.29	0.705
Gender (Male/female)	14/16	14/16	1
ASA (II/III)	26/4	25/5	0.718
Duration of surgery (min)	140.1 ± 55.3	126.73 ± 61.06	0.378
Intraoperative blood loss (mL)	23.63 ± 20.04	30.19 ± 44.18	0.405
Intraoperative infusion volume (ml)	738.89 ± 255.83	816.67 ± 286.56	0.378
Length of hospital stay (days)	11.77 ± 4.15	14.67 ± 6.91	0.054

Data are mean ± standard deviation. ASA, American Society of Anesthesiology.

### 3.3 VAS and BCS scores

**FIGURE 2:**
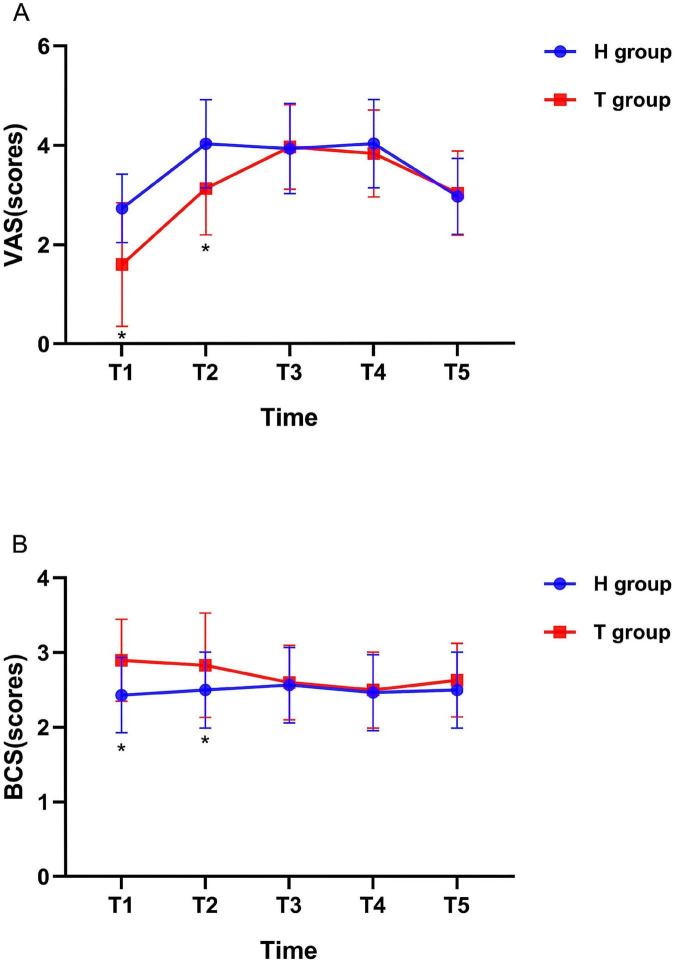
Variables reflecting the VAS and BCS scores of the two groups at different time points. *n* = 30 for each group. **(A)** Visual analog scale; **(B)** bruggrmann comfort scale. *P < 0.05 vs. H group.

As displayed in Figure 2, compared to the H group, patients in the T group showed a decrease in VAS scores and an increase in BCS scores at T1 and T2 (*P* < 0.05). But there was no significant difference in VAS and BCS scores between the two groups at T3, T4 and T5 (*P* > 0.05).

### 3.4 Opioid dosage

**FIGURE 3:**
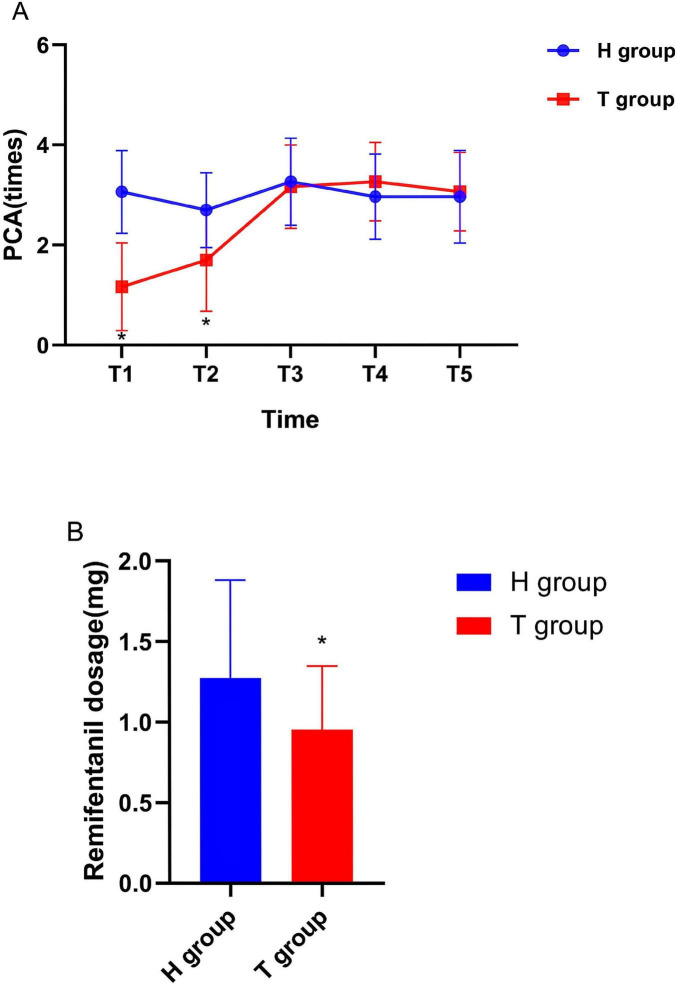
**(A)** PCA times of the two groups at different time points. **(B)** shows the dosage of remifentanil used during surgery. *n* = 30 for each group. PCA, Patient Controlled Analgesia. *P < 0.05 vs. H group.

As displayed in Figure 3A, compared to the H group, the PCA frequency of the T group was significantly reduced at T1 and T2 (*P* < 0.05). But there was no significant difference in PCA frequency between the two groups at T3, T4 and T5 (*P* > 0.05). As displayed in Figure 3B, the dosage of remifentanil in T group during surgery was significantly lower than that in H group (*P* < 0.05).

### 3.5 The inflammatory reaction index

As shown in Table 2, before operation, there was no significant difference in CRP, TNF-α and IL-6 levels between the two groups (*P* > 0.05); The levels of CRP, TNF-α and IL-6 in the two groups after operation were higher than those before operation, and the levels of CRP, TNF-α and IL-6 in T group were lower than those in H group (*P* < 0.05).

**TABLE 2:** Comparison of inflammation between two groups.

Index	Time	H group(*n* = 30)	T group(*n* = 30)	*t*	*P-value*
CRP (mg/L)	Preoperative	1.95 @ 1.01	2.12 @ 1.11	-0.617	0.54
	1 days after operation	62.09 @ 3.76^#	46.16 @ 5.15^#*	13.68	<0.001
IL-6 (pg/ml)	Preoperative	82.16 @ 4.51	82.63 @ 4.02	-0.419	0.677
	1 days after operation	122.74 @ 4.70^#	109.77 @ 4.05^#*	11.448	<0.001
TNF-α (pg/ml)	Preoperative	46.04 @ 3.13	45.13 @ 3.23	1.103	0.275
	1 days after operation	60.13 @ 3.32^#	53.61 @ 3.34^#*	7.584	<0.001

^#^*P* < 0.05 vs. baseline; *P < 0.05 vs. H group.

### 3.6 Complications

As shown in Table 3, there was no significant difference in the bradycardia, hypotension, nausea and vomiting between the two groups (*P* > 0.05). The first time patients in T group got out of bed after surgery was earlier than that in H group (*P* < 0.05).

**TABLE 3:** Comparison of complications between two groups.

Index	H group(*n* = 30)	T group(*n* = 30)	*P-*value
**Adverse reaction**			
Bradycardia [*n* (%)]	0 (0.00%)	1 (3.7%)	0.317
Hypotension [*n* (%)]	0	0	0
Nausea [*n* (%)]	2 (6.67%)	3 (10%)	0.642
Vomiting [*n* (%)]	0	0	0
First time of getting out of bed activity after surgery (h)	24.27 ± 2.86	16.7 ± 2.97*	<0.001

*P < 0.05 vs. H group.

## 4 Discussion

The research results showed that the VAS score significantly decreased and the BCS comfort score increased in T group at 4 and 8 h after surgery (*P* < 0.05). This proves that the multimodal analgesia of thoracic paravertebral nerve block combined with intravenous analgesia has a significantly higher analgesic effect within 8 h than the use of hydromorphone alone for patient-controlled analgesia. The thoracic paravertebral space is connected outward to the intercostal space, inward to the epidural space through the intervertebral foramen, and can be connected to the contralateral paravertebral space through the vertebral body and epidural space. Within this gap, there are intercostal nerves, dorsal branches of spinal nerves, communicating branches, and sympathetic chains. Therefore, injecting local anesthetics within the gap can achieve ipsilateral regional anesthesia (32). It not only inhibits the release of inflammatory factors caused by pain stimuli, but also prevents increased sensitivity of surrounding neurons and the central nervous system to pain.

In the experiment, the amount of remifentanil used during surgery in T group was significantly reduced compared to H group (*P* < 0.05). At TI and T2, the number of PCA iterations in T group was less than that in H group (*P* < 0.05), while at T3, T4 and T5, the difference in PCA iterations between the two groups disappeared (*P* > 0.05). Perhaps due to the weakened efficacy of ropivacaine in group T after 8 h of blockade, pain relief gradually relies on the analgesic effect of opioid drugs. This may be related to the duration of nerve block effect of disposable ropivacaine (33). So, in the future, we can continue to study whether continuous thoracic paravertebral nerve block can further improve the early recovery of surgical patients by prolonging postoperative analgesia time. In addition, it is worth exploring the feasibility of using ultrasound-guided thoracic paravertebral nerve block before and after surgery to prolong the duration of postoperative analgesia when continuous medication cannot be met.

Ropivacaine, a commonly used long-acting local anesthetic in clinical practice, belongs to the amide class of drugs. It has the advantages of motor sensory dissociation and lower cardiac toxicity and neurotoxicity compared to other local anesthetics. The use of ropivacaine for nerve block anesthesia is safer and has less impact on postoperative movement (34). At present, there is no clear concentration range for the application of ropivacaine in nerve block. At present, most single dose ropivacaine drugs used for nerve block in clinical practice have a drug capacity of 20 ml–40 ml and concentration of 0.25% to 0.5% (35, 36). Griffiths et al. (37) suggested that the concentration range of ropivacaine used for nerve block should be between 0.2% and 0.5% and the total capped dose should not exceed 210 mg. The concentration and dosage of ropivacaine referred to in this study are also within this range.

Our previous research has shown that both direct surgical trauma and pain caused by trauma can trigger inflammatory stress in the body, releasing a large amount of chemical neurotransmitters and inflammatory factors that delay postoperative recovery in patients (38). The detection of serum inflammatory factor levels plays an important reference value in determining the degree of stress response in the body. Studies have shown that the expression level of inflammatory factors is positively correlated with the size of surgical trauma and the degree of stress response (39). Therefore, it is important to effectively alleviate perioperative inflammatory responses for patients following surgery, especially major surgery. The experimental analysis results show that there was no statistically significant difference in preoperative serum inflammatory factor levels between the two groups (*P* > 0.05). Two groups showed a significant increase in postoperative inflammatory factors compared to preoperative levels (*P* < 0.05). This study further confirms that surgical related tissue damage can induce stress responses in the body and further promote the release of inflammatory cytokines during the perioperative period, including CRP, IL-6, and TNF-α (40). Compared with H group, the postoperative serum levels of IL-6, TNF - α, and CRP in T group were lower (*P* < 0.05). This indicates that thoracic paravertebral nerve block reduces the inflammatory response caused by perioperative surgical trauma. The lower levels of plasma CRP, IL-6, and TNF-α in T group compared to H group after surgery may be related to pain relief (41).

Interleukin-6 is not only a major pro-inflammatory cytokine, but also a pain sensitizer. The massive release of IL-6 can increase the sensitivity of central and peripheral nerves to pain. It starts to increase 2 h after the inflammatory response and reaches its peak at 24 h (42). TNF - α is a pain mediator secreted by macrophages that triggers inflammatory responses, exacerbates tissue damage, rapidly increases in the early stages of stress, and reaches its peak at 12 h (43). CRP, a clinically sensitive indicator of the degree of inflammatory response, is an acute reactive protein produced by the liver that begins to increase 6–8 h after the body is subjected to traumatic stress and reaches its peak 24–48 h (44). By weighing the time required for the three inflammatory factors mentioned above to reach peak levels, this experiment selected serum samples collected 24 h after surgery for testing. The results of this study suggest that it may be due to the analgesic mode of T group that achieving sufficient pain relief for patients during the perioperative period, inhibiting the body’s pain stress and pain sensitization, thereby weakening the effect of inflammatory response.

There was no statistically significant difference in the incidence of postoperative bradycardia, hypotension, nausea and vomiting between the two groups (*P* > 0.05). The first time T group got out of bed was earlier than H group (*P* < 0.05). The hospitalization time in T group was shorter than that in H group, but there was no statistical significance (*P* > 0.05). The anti-inflammatory and analgesic effects of the combination therapy can reduce postoperative pain and the need for opioid drugs, which reduces adverse reactions related to opioid drugs, such as postoperative nausea and vomiting. In addition, it can also reduce hospitalization time to a certain extent. Danish physician Kehlet proposed the concept of Enhanced Recovery After Surgery (ERAS) that further shifts the focus of various clinical surgical systems beyond surgical operations (45). The philosophy of ERAS is to optimize a series of perioperative management measures based on evidence-based medicine in order to minimize adverse reactions caused by surgery and accelerate patient recovery. The application of thoracic paravertebral nerve block under the ERAS concept can effectively alleviate postoperative pain, reduce perioperative opioid use, accelerate patient recovery, and improve patient satisfaction in thoracoscopic lobectomy surgery (16, 46).

The limitations of the research was that the sample size of this study is relatively small and there has been no collaboration with other institutions. In the future, we expect to conduct large-scale, multicenter clinical studies. This study only used the application effect of local anesthetics at a single concentration and did not compare the application of drugs at different concentrations. This study only conducted a single serum test and did not investigate the levels of inflammatory response factors at different times throughout the perioperative period.

## 5 Conclusion

In summary, ultrasound-guided thoracic paravertebral nerve block combined with hydromorphone in thoracoscopic lobectomy surgery can have a good analgesic effect, reduce the amount of opioid drugs used during the perioperative period, alleviate the body’s inflammatory response, promote rapid recovery, and have good application value.

## Data Availability

The raw data supporting the conclusions of this article will be made available by the authors, without undue reservation.
